# Determining adult type 2 diabetes-related health care needs in an indigenous population from rural Guatemala: a mixed-methods preliminary study

**DOI:** 10.1186/1472-6963-12-476

**Published:** 2012-12-22

**Authors:** Anita Chary, Miranda Greiner, Cody Bowers, Peter Rohloff

**Affiliations:** 1 Wuqu’ Kawoq, 2 Calle 5-43 Zona 1, Santiago Sacatepéquez, Guatemala

**Keywords:** Type 2 diabetes, Indigenous health, Guatemala, Non-communicable disease

## Abstract

**Background:**

In Guatemala, diabetes is an emerging public health concern. Guatemala has one of the largest indigenous populations in Latin America, and this population frequently does not access the formal health care system. Therefore, knowledge about the emergence of diabetes in this population is limited.

**Methods:**

Interview participants (n=23) were recruited from a convenience sample of indigenous adults with type 2 diabetes at one rural diabetes clinic in Guatemala. A structured interview was used to assess knowledge about diabetes and its complications; access to diabetes-related health care and treatment; dietary and lifestyle changes; and family and social supports for individuals living with diabetes. Interviews were supplemented with two group interviews with community leaders and health care providers. Thematic analysis was used to produce insights into diabetes knowledge, attitudes, and practices. In addition, a chart review of the clinic’s electronic medical record identified all adult patients (n=80) presenting in one calendar year for a first-time diabetic consultation. Sociodemographic and clinical variables were extracted and summarized from these records.

**Results:**

Salient demographic factors in both the structured interview and chart review samples included low educational levels and high indigenous language preference. In the interview sample, major gaps in biomedical knowledge about diabetes included understanding the causes, chronicity, and long-term end-organ complications of diabetes. Medication costs, medical pluralism, and limited social supports for dietary and lifestyles changes were major practical barriers to disease management. Quantitative data from medical records review revealed high rates of poor glycemic control, overweight and obesity, and medication prescription.

**Conclusions:**

This study provides a preliminary sketch of type 2 diabetes in an indigenous Guatemalan population. Combined qualitative and quantitative data point towards particular needs for implementation and future research, including the need to address gaps in diabetes knowledge, to improve social support systems, and to address the cost barriers associated with disease treatment.

## Background

The growing burden of non-communicable diseases (NCDs) in low and middle-income countries is one of the most important global health challenges today. In 2008, 63% of all global deaths were due to NCDs [1]. Nearly 350 million people worldwide have diabetes; of these, 80% live in low- and middle-income countries, especially China, India, and Latin America [2,3]. In these countries, rates of diabetes are rising in both urban and rural contexts [4]. The number of deaths from diabetes worldwide is projected to double in the next 20 years [3].

### Diabetes in Latin America and Guatemala

Data on the burden of diabetes in Latin America as a region are limited and heterogeneous, although new data is now emerging from coordinated efforts, such as the Pan American Health Organization-sponsored Central American Diabetes Initiative, which has found a combined prevalence in Central American urban populations of 8.5% [5]. For comparison, an earlier study in Mexico City found a rate of 10.7% [6], and rates in urban South American populations have ranged from 6.5-7.6% [7-10].

Guatemala is a Central American country with approximately 15 million inhabitants. Guatemala is following global and regional trends towards an increasing burden of diabetes, driven in part by a rise in the prevalence of sedentary lifestyle, increased urbanization, and the erosion of traditional agricultural lifestyles [11-13]. The last thirty years have seen steady increases in mean body mass index in both Guatemalan men and women [14]. A recent statistical model estimated an increase in the prevalence of diabetes in men from 8.9% to 11.5% and in women from 8.0% to 14.0% over the last three decades [2]. In a recent study in an urban population near the capital of Guatemala City, the overall prevalence of diabetes was 8.4% [12].

### The role of indigeneity

One demographic feature that makes Guatemala fairly unique in Latin America is the high percentage of the population who are indigenous (66% of total population) [15]. Other countries in Latin America with a similarly large indigenous population include Bolivia (71%), Peru (47%), and Ecuador (43%) [15]. Around the world, indigenous people routinely suffer from health care disparities, which are only in part explained by the fact that they tend to inhabit rural areas with limited health care infrastructure [15,16]. In Guatemala also, the most recent national health surveys demonstrate large disparities in many health indicators for the indigenous population [17].

The historical reasons for health care disparities for indigenous populations in Guatemala are complex. They include centuries-old policies of social and economic marginalization dating back to the times of the Spanish Conquest and the recent conclusion, in 1996, of a violent civil war, which disproportionately targeted indigenous communities [18]. Although a recent major post-war initiative by the government of Guatemala has resulted in the expansion of basic health care services to more than 3 million indigenous inhabitants [19], mistrust and underutilization of this system remains high, in no small measure due to cultural and linguistic barriers [20]. Furthermore, this system remains severely under-resourced. For example, more than 80% of physicians in Guatemala are concentrated in the capital city, meaning that although the ratio of physicians to inhabitants is 10 per 10,000 nationwide, in many indigenous areas the effective ratio is closer to 1 per 10,000 [21]. Indeed, in some regions with the highest indigenous population, more than 80% of government physician posts are unfilled [22].

With respect to the prevalence of NCDs, including diabetes and associated risk factors such as obesity, there is minimal data on the indigenous population of Guatemala. The bulk of systematic investigations of NCDs and NCD risk factors in rural Guatemala have been conducted in non-indigenous communities [11,23-26]. At the same time, anecdotal data from the Guatemalan Ministry of Health (MOH) suggest a trend towards increasing diagnosis of diabetes in indigenous areas [27].

The MOH has developed guidelines for the detection and treatment of diabetes, which are meant to be broadly applicable, even in rural indigenous settings [28]. However, in reality, resources for diabetes treatment are often unavailable in these areas. In addition to the issues of limited infrastructure and mistrust mentioned above, another factor that drives lack of access is the high cost of medications and frequent breaks in the medication supply line. For example, a recent study has documented high rates of unavailability of medications on the WHO essential medications list across a range of public and private outlets [29]. Another report has demonstrated that supply line breaks for medications are endemic throughout the formal health care system, but especially affect the programs for extending health coverage into rural indigenous areas [30].

### Aims and scope of this research

In order to develop effective diabetes programs in rural, indigenous areas of Guatemala, qualitative research about the diabetes-related knowledge, attitudes, and practices in this population is needed. At the same time, quantitative research is needed in order to define the demographic characteristics of the population with diabetes, explore the availability and effectiveness of therapeutic interventions, and describe patterns of modifiable disease risk factors. In this paper, we use mixed methods to achieve these two aims. First, we use qualitative methods (structured individual and group interviews) to explore knowledge, attitudes, and practices about as well as experiences with diabetes in a rural indigenous sample population. Second, we conduct a chart-review of type 2 diabetic patients presenting to one rural nonprofit clinic during a single calendar year, in order to extract quantitative sociodemographic data, as well as data on common modifiable disease risk factors, glycemic control, and medication prescription.

## Methods

### Institutional context

Research was conducted through Wuqu' Kawoq, a nongovernmental health organization working in central Guatemala with rural indigenous communities to develop chronic disease management programs. Research methodology underwent appropriate review by Wuqu' Kawoq's Institutional Review Board.

### Overview of methods

This study employed a mixed-methods approach, collecting qualitative data through structured individual interviews and group interviews, as well as quantitative data through review of medical records. Participants in the study were a convenience sample of adult patients (>18 years of age) with type 2 diabetes who attended Wuqu’ Kawoq’s clinics in the central highland municipalities of Santiago Sacatepéquez (*population* 28,000*)*, San Juan Comalapa (*pop.* 35,000), and Tecpán (*pop.* 60,000), as well as the piedmont municipality of San Pablo Jocopilas (*pop.* 20,000). In each of these sites, the indigenous population constitutes the majority and many residents speak Kaqchikel or K’ichee’ (two of the most widely-spoken Mayan languages) exclusively or in addition to Spanish.

Formal health services are available in each community. All four communities contain MOH health posts with full-time physician staff offering a range of services, including obstetrical care and care for NCDs. In addition, Tecpán contains a small MOH-funded satellite hospital. Both Tecpán and San Juan Comalapa are targeted for additional preventative health care services via the MOH’s rural health care expansion initiative, whereas Santiago Sacatepéquez and San Pablo Jocopilas are not. All four towns are within a 90-minute drive from a national hospital. Finally, all four communities are the targets of a range of development activities sponsored by nongovernmental organizations, including preventative and curative health care services.

### Structured interviews

In each site, a local indigenous staff member was asked to contact all diabetics receiving health care through Wuqu’ Kawoq programming to inquire about interest in participating in the study. Authors (AC, MG) followed up with those patients who were willing and available to be interviewed. In all, structured interviews were conducted with 23 diabetic patients (San Juan Comalapa, n=6; San Pablo Jocopilas, n=5; Santiago Sacatepéquez, n=7; Tecpán, n=5). Most interviews were conducted in Kaqchikel Maya (n=15), although some were conducted in Spanish (n=8), depending on the preference of the informant. The authors were proficient in both Kaqchikel and Spanish, and they were also accompanied by a female staff member of Wuqu’ Kawoq, previously unknown to the participants, who was a native Kaqchikel speaker and was able to clarify questions or responses as necessary. Verbal informed consent was obtained from all participants, and no personal identifying data were recorded. Interviews were conducted within Wuqu’ Kawoq clinical facilities (n=11) or patient homes (n=12), depending on interviewee preference, from June through August of 2010.

The structured interview consisted of 71 items: 20 open-ended questions about personal experiences with diabetes (e.g. “How did you find out you had diabetes?”), and 51 closed-ended questions concerning general knowledge, dietary practices, and demographic information (e.g. “Do you have a family member with diabetes?”). Interview items were structured into 5 major thematic areas: manner of diagnosis, knowledge of disease process, treatment, diet, and social supports. Interview times ranged from 45 to 240 minutes.

### Group interviews

Individual structured interviews were supplemented with one group interview with 3 health care providers (one midwife, one auxiliary nurse, and one community health worker) in San Juan Comalapa and one group interview with 3 non-diabetic community leaders in San Pablo Jocopilas. Members of each of these groups heard about the study from interviewees and requested to participate in order to give their perspectives. After individual interviews were completed in each location, one investigator (AC) held these group interviews, soliciting opinions about the themes that had surfaced in the individual interviews. Conducting these group interviews allowed for observation of consensus and disagreement regarding the investigators’ preliminary interpretations of themes from the individual interviews. In this way, the group interviews helped to guide data analysis. The group interview in San Juan Comalapa lasted 60 minutes, and the group interview in San Pablo Jocopilas lasted 120 minutes.

### Qualitative data analysis

Individual and group interviews were not recorded, due to the preferences of the informants. Therefore, extensive notes were taken throughout the interactions by two authors (AC and MG). Subsequently, both authors performed a thematic analysis, coding the data around the 5 major thematic areas (diagnosis, knowledge, treatment, diet, social supports) and cross-checking for accuracy. One final author (PR) reviewed coded data and resolved contested issues.

### Medical records review

Chart review of Wuqu' Kawoq's electronic medical record was conducted. Diabetic patients were identified from the electronic medical record by searching for the concepts “diabetes,” “glycosylated hemoglobin,” “blood glucose,” and the three medications metformin, glibenclamide, and insulin. These three medications were chosen because they are the only three diabetes medications prescribed in the clinic. The date range of the records search was 01/01-12/31/2011. This search generated a total of 354 database hits, which were then manually reviewed to generate a list of the medical records of patients presenting for their first diabetes consultation in the clinic. The list was further refined to include only adult patients (≥ 18 years of age) and to exclude those with a diagnosis of type 1 diabetes. In all, 80 patients with first-time type 2 diabetes consultations within the specified time range were identified. For each patient, the following data was extracted from this first clinical encounter and from review of their demographic face sheet: age, sex, preferred language, educational level, tobacco use, alcohol use, years since diagnosed with diabetes, body mass index, systolic and diastolic blood pressure, glycosylated hemoglobin, medication prescriptions, and creatinine. After completion of data extraction, linkages to health records were immediately destroyed.

### Quantitative data analysis

Data were entered into a spreadsheet and then imported into a statistical software package (STATA, College Station, TX), which was used to generate the descriptive statistics presented in Table 1. Additionally, STATA was used to perform tests for significance for differences in sociodemographic variables of the structured interview cohort vs. the medical chart review cohort. A two-tailed Student’s t-test was used for continuous parametric variables (age, duration of diabetes); Fisher’s exact test was used for categorical variables (gender, language); and the Wilcoxon rank-sum test was used for nonparametric variables (years of schooling).

**Table 1:** Characteristics of adults with type 2 diabetes identified by medical chart review

**Characteristic**	**Total n=80**
Age – yrs (n=80)	54.9 ± 12.7
Female – % (n=80)	85
Language preference – % (n=80)	
Kaqchikel Mayan	75.7
Spanish	21.3
K’ichee’ Mayan	3.0
Primary school education – yrs (n=63)	
Median	2
Interquartile range	0-4
Tobacco use – % (n=80)	0
Alcohol use – % (n=68)	4.4
Time since diagnosis – yrs (n=63)	
Median	6
Interquartile range	4-11
Body mass index (n=63)	29.1 ± 4.3
Body mass index ≥ 25 – %	88.9
Body mass index ≥ 30 – %	38.1
Blood pressure – mmHg (n=78)	
Systolic	120.1 ± 16.3
Diastolic	75.7 ± 10.0
Systolic BP ≥ 140 mmHg – %	12.8
Diastolic BP ≥ 90 mmHg – %	17.9
Hypertension – %	14.1
Glycosylated hemoglobin – % (n=79)	8.9 ± 2.1
Glycosylated hemoglobin < 7% – %	19.0
Glycosylated hemoglobin < 8% – %	43.0
Insulin prescription – %	7.5
Metformin prescription – %	93.8
Sulfonylurea prescription – %	64.1
Creatinine – mg/dl (n=62)	0.94 ± 0.41
Creatinine ≥ 1.5 mg/dl – %	3.2

In Table 1, alcohol use percentage reflected current usage at the time of chart review, whereas tobacco use was recorded as ever having used tobacco. The percentage of patients with hypertension was calculated by combining both the number of patients meeting criteria for systolic (≥140 mmHg) or diastolic (≥90 mmHg) hypertension with the number of patients with a prescription for an antihypertensive medication, regardless of recorded blood pressure values. The cut-off for BMI ≥ 25 (overweight) included also the proportion of patients with a BMI ≥ 30 (obese).

## Results

### Findings of structured and group interviews

Basic demographic information of participants in structured interviews is summarized in Table 2. Thematic analysis of the 23 structured interviews are summarized here.

**Table 2:** Demographic characteristics of structured interview participants

**Characteristic**	**Total N=23**
Age – yrs	61.4 ± 13.8
Female – %	78
Language preference – %	
Monolingual Mayan	26
Monolingual Spanish	9
Bilingual Mayan/Spanish	65
Education – yrs	
Median	1
Interquartile range	0-6
Time since diagnosis – yrs	
Median	5
Interquartile range	3-14

### Diagnosis

A minority of informants had prior knowledge of diabetes before their diagnosis. Some informants did report that, based on their symptoms, they were encouraged to seek a blood glucose test by a more knowledgeable family member or acquaintance. For example, an older man described that upon beginning to experience severe thirst and dry mouth while harvesting crops, the landowner of the fields he worked instructed him to get his “sugar” checked. Nevertheless, a significant proportion of diagnoses of diabetes were incidental (26%, *n* = 6). For example, one informant noted that she went to a health center for a fever and cold, with suspicions that it was not a “simple cold” (“*gripe sencilla*”)*,* and remarked that on receiving her laboratory exam results, it was diabetes just as she had thought (“*cabal fue diabetes*”).

### Symptoms and sequelae

Most informants were familiar with symptoms of hyperglycemia, such as dizziness, thirst, frequent urination, and weight loss. However, fewer were aware of long-term sequelae of diabetes, such as neuropathy, cardiovascular disease, nephropathy, and other forms of end organ damage. Only those patients who had experienced diabetes-related vision problems or vision loss were aware of the effects of diabetes on vision; these patients opined that diabetes is a “very serious” condition (“*muy grave,*” “*yalan k’ayew*”).

### Causation

Informants most often interpreted the onset of the disease as the result of a strong emotional experience or *susto (*“fright”)*.* Several interviewees noted that the emotional stress of enduring violence during the civil war brought on their diabetes (“*por miedo de la violencia” –* “because of fear of the violence”). During group interviews, community leaders echoed sentiments that emotions can “accumulate in the body” to toxic levels, while health workers underscored the commonality of these attitudes (“*así dice la gente aquí”* – “that’s what people here say”). Additionally, several patients described that familial conflicts, such as abusive relationships with husbands and in-laws or community gang violence, caused severe bodily stress that could provoke diabetes (“*Xe violencia, xe azúcar”* – “Nothing but violence, nothing but diabetes”).

Several participants felt that diabetes was more prevalent today than when they were younger (“*Wakamin, xäb’achike winäq. Ojer kan más manäq*” – “Today, any person [can get it]. In the old days, not so much”). Community leaders participating in one of the group interviews shared this attitude, stating that in the “old days,” people ate natural foods and worked hard in the fields, whereas today the food is not the same and many people go blind with diabetes. On the other hand, even though most informants had relatives who were also diabetic (83%, n=19), few were aware of the hereditary component of type 2 diabetes.

### Cure

The majority of informants (70%, n=16) felt that diabetes could not be cured: "*No se cura, sólo se controla" - “*It can't be cured, only controlled*”.* However, the remainder did feel that diabetes could be cured, or at least that adherence to a life-long regimen of control was not necessary. Some patients referred to faith and God’s ability to work miracles as possible cures for diabetes (“*Xe Dios retaman,*” “*Xe Dios ntikïr*” – “Only God knows,” “Only God can [cure it]”). Additionally, several interviewees reported that they had followed a diabetic diet or taken medications for a few months, but had discontinued treatment once they began to feel better. One diabetic patient, who had lived with the disease for more than a decade, joked that most diabetics do not believe in the disease until they see it, and they stop believing it once they can no longer see it (“*no lo creen hasta que lo miren, y dejan de creerlo cuando ya no lo pueden ver*”). Participants in our group interviews also reflected that most diabetics were adherent to their regimens only after “learning their lesson” by trying to discontinue medications multiple times and experiencing severe symptom relapse.

### Current treatment methods

Most informants endorsed an eclectic combination of pharmaceuticals, medicinal plants, diet, and prayer to treat their diabetes. Seventy eight percent (*n* = 18) reported taking pharmaceuticals at some point, and 48% (*n* = 11) stated that they currently took pharmaceuticals on a daily basis. Those who had experienced more severe symptoms related to hyperglycemia were more likely to be taking pharmaceuticals on a daily basis, while those who felt less bothered by their hyperglycemia were more likely to endorse an intermittent regimen, usually because they became tired of taking the medications (“*xikos yan*,” "*se aburre uno*" - “I got tired,” “I got sick of it”).

### Costs

Informants routinely reported that the cost of medications was a major barrier to diabetes treatments. Most reported spending thousands of quetzales out of pocket for diabetes treatments. For comparison, the mandated minimum daily wage for agricultural workers in Guatemala is around 64 quetzales (~ 8 USD), although most indigenous workers make considerably less, due to high levels of participation in the informal economy [31]. Insulin in particular was reported as expensive, with a single vial often costing as much as 250 quetzales (~ 30 USD). One woman reported that previously her private physician had prescribed two pills, metformin and glibenclamide. While she had taken glibenclamide on a daily basis (“*diario xintïj*” – “*I took it daily*”) because it was more affordable, she supplemented it with metformin irregularly, only when household income allowed (“*k’o b’ey manäq*” – “*there are times that I don’t [take metformin]*”).

Medical consultations were also viewed as prohibitively expensive. Several informants complained about the pointlessness of visiting a doctor, whether in the public or private sector, who would charge a consultation fee and offer nothing in return except a prescription for an expensive medication. Interestingly, for several informants treatment at the hands of naturopaths or traditional practitioners resulted in costs several-fold higher than those associated with allopathic treatments.

### Medical pluralism and the complexity of treatment

Most patients reported considerable confusion regarding the various treatment venues available to them, including pharmacies, allopathic medical practitioners, traditional practitioners, nutritionists, and religious services. Combined with the prohibitive costs of many treatment modalities (see above), this often resulted in prolonged delays in accessing definitive allopathic treatments and multiple relapses in glycemic control. This situation is best illustrated by consideration of a case study derived from a single structured interview participant, which illustrates the confluence of factors that impact diabetes care.

This 55 year-old Kaqchikel-speaking woman began to experience a number of vague constitutional symptoms such as fatigue and dizziness. Family and friends advised her that these symptoms might represent diabetes, and she sought formal diagnosis at a private laboratory that confirmed severe hyperglycemia. She initially sought treatment with a local private physician and her symptoms resolved. However, the high cost of treatment and her fluctuating financial security resulted in several “relapses” during which she took no medications at all, only to resume treatment when she had saved up more money. Increasingly desperate because of worsening symptoms and the cost of treatment, she then spent several years seeking out treatment from multiple parallel sources, including the MOH post, an additional private medical clinic, an herbal medicine practitioner and, most alarmingly, periodic injections of insulin in a local pharmacy whenever she was “feeling really bad.” She eventually made the acquaintance of one of our community health liaisons, which led her to seek a consultation for disease management with us. The entire elapsed time was approximately 10 years.

During group interviews, participants opined that due to distrust, economic struggles, and perceived inefficacy of treatment, many diabetics take complicated treatment paths and lack continuity of care with one provider. Health workers described that patients often resorted to herbal home remedies during times of economic duress until they could once again afford expensive pharmaceutical prescriptions, medical consultations in the private sector, or herbal treatments with naturopaths.

### The diabetic diet

When asked what diet a diabetic patient should follow, interview participants most commonly mentioned decreasing sugar or fat intake or portion size, eliminating soft drinks, or increasing fruit and vegetable consumption; however, 30% (n=7) felt no dietary changes were necessary. When asked what barriers they encountered in adhering to a diabetic diet, they also offered numerous insights.

First, food was bland, flavorless, and no longer enjoyable when following a diet. One elderly woman’s statement was emblematic of the sentiments of many interviewees: *"Si como, me muero. Si no como, me muero. Mejor gozo" - "If I eat, I die. If I don't eat, I die. Better to enjoy [life]."* Several reported feeling sad as they walked through markets, seeing foods that they used to be able to eat. Interviewees also reported constant hunger while following the diet, associated in particular with restricting corn tortilla intake (which are a cheap source of calories in rural Guatemala and are often used to compensate for scarcity of other foods). Many reported that their stomachs did not “fill up” when they restricted tortilla consumption (“*Man ninöj ta nupam,”* “*Así no se llena*” – “My stomach doesn’t fill up,” “You can’t get full that way”) or that they no longer had the energy to perform household chores or visit the market (“*Ri nufuerza manäq*” – *“I don’t have any strength*”).

Informants often cited financial costs associated with dietary diversity, especially with consuming higher quantities of fruits and vegetables. As one woman offered: “*Röj pobre. Man k’o ta nupwaq, ronojel jotöl.” –* “*We are poor. I don’t have money, and everything is expensive.*” While this woman is a spinach vendor, she and her family sell their produce on a daily basis rather than keeping it for their own consumption. Indeed, among interviewees with access to land, most reported growing a variety of products but retaining only corn and beans for their own households.

Many diabetics noted logistical difficulties of eating differently than the rest of the family. Notably, this theme did not surface during interviews with men or with women who were considered the “woman of the house”, as these household members could typically prepare food for themselves or instruct those in charge of meal preparation about their dietary needs. On the other hand, women who lived in the homes of their in-laws did not occupy a household position prestigious enough to merit individualized attention to meal preparation. One elderly woman remarked that she had started following the diet more closely when she and her husband moved out of their son and daughter-in-law’s house.

An additional difficulty occurred during attendance at parties, celebrations, weddings, and other social events, where informants did not feel at liberty to refuse food offered to them. During the first group interview, health care providers compared diabetics to alcoholics who believe that “one little sip” will not hurt, stating that diabetics renege on their diets during celebrations due to erroneous assumptions that “one little piece” here and there will not affect their wellbeing overall. One community health worker noted that “one little piece” quickly turns into a plateful of unhealthy foods.

### Social supports

Family members played a significant role in informants' therapeutic and dietary choices. Patients' children and male patients’ wives were often observed encouraging adherence to a healthier diet, providing reminders to take medications, and purchasing special foods. Children bilingual in Kaqchikel and Spanish played a particularly important role in healthcare coordination for monolingual Kaqchikel relatives with diabetes.

However, other diabetic informants reported feeling ostracized or resented by their families, especially due to the extra burden of preparing special meals. Some female informants reported feeling socially vulnerable due to their diabetes; for example, one expressed her fears that diabetes would make her infertile and cause her husband to abandon her. In many instances, informants reported that their family members did not take their dietary restrictions seriously and constantly tempted them with prohibited foods. An elderly man explained his son persisted in “showing him affection” by buying him soft drinks on the way home from work. Some interviewees, who were widows or widowers living without their children or other family members, also lacked social support because of the absence of family members. One widow reported that she used to take medications regularly, but once her youngest son moved out of the house, she began to feel sad, “forgot” to take medications, and found it more difficult to adhere to her diet. Participants in the group interview with health care providers offered similar accounts about the role of family in shaping diabetics’ experiences, and one stated that family is the “biggest problem” (“*el problema más grande*”) for diabetics.

### Findings of medical chart review

As described above under “Methodology” we used a review of electronic medical records to identify 80 unique adult patients with type 2 diabetes who presented for a first time diabetes consultation in 2011. Demographic and clinical information on these patients are summarized in Table 1. Data on all variables was not available for all patient records, and this information is also reflected in Table 1.

The key demographic findings of this chart review include the strong preference for use of Mayan languages and low levels of educational attainment. Among modifiable risk factors, tobacco and alcohol use were exceedingly low. On the other hand, 89% of patients had an abnormal BMI (BMI ≥ 25), and 14% had hypertension. In terms of diabetes control, only 19% and 43% of patients had a glycosylated hemoglobin measurement of less than 7% or 8%, respectively. Finally, as an indicator of end-organ damage, we analyzed serum creatinine measurements, finding that 3.2% of patients had a serum creatinine of ≥ 1.5 mg/dl.

Since our study involves two separate sampling methods of the cohort of individuals with type 2 diabetes in the clinic (structured individual interviews versus chart review), we performed statistical tests of significance for differences between basic sociodemographic variables for these two samples. The mean age of the structured interview cohort was significantly higher than the chart review cohort (p=0.04), but other variables, including gender (p=0.53), years of education (p=0.47), duration of diabetes (p=0.96), and language preference (p=0.33), were not significant.

## Discussion

Given the near complete lack of data on type 2 diabetes in rural indigenous Guatemala, we have undertaken here a mixed-methods study involving both structured and group interviews to assess knowledge, practices, and attitudes regarding diabetes, as well as a medical chart review to collect information on disease characteristics and modifiable risk factors in this setting. Several important themes emerge from our results.

First, our thematic analysis of responses from interview participants showed several areas of deficient biomedical knowledge and diabetes-related practices. For example, a significant proportion of our respondents were unaware of the long-term end-organ complications of diabetes, of the disease’s chronicity, or of the role of heredity. Diabetes diagnosis was often incidental, and adherence to daily medication regimens or to dietary restrictions was a struggle. Additionally, the majority of patients attributed diabetic causality to stress or fright. Comparison of our findings with those of a recently published results of a large NCD survey of urban non-indigenous Guatemalans [12] is instructive, because that survey showed very similar deficiencies in knowledge and practice, especially in the areas of incidental diagnosis, diet, and medication adherence. This indicates that the problem areas identified in our qualitative survey are not necessarily unique to the indigenous population, nor even to Guatemala. Indeed, gaps in diabetes knowledge are universal in both developed and developing countries alike, across a broad cultural spectrum [32,33]. Furthermore, attribution of diabetes to stress is a very common causal point of view in most Latin American populations [34].

Despite these similarities, it is important to focus on the specific sociodemographic characteristics of the indigenous population that are likely to impact the effectiveness of educational programming and to require contextualization. In particular, in both our structured interview and chart review samples (Tables 1 and 2), a strong language preference for a Mayan language was exhibited, and educational levels were simultaneously extremely low. Although opportunities for use of first languages in public education have improved significantly in recent years for indigenous Guatemalans, programming that addresses the health needs of indigenous adults will continue to need to grapple with a pre-civil war historical legacy of linguistic marginalization through obligatory Spanish-only education, which resulted in abysmal rates of educational achievement [35]. Similar linguistically-conditioned barriers to diabetes care have been observed in other indigenous groups around the world [36-38].

Another important issue identified in our interviews was the high cost of diabetes treatments, leading in many cases to multiple cycles of treatment relapse. For example some informants reported spending as much as 250 quetzales on a vial of insulin, which is approximately equivalent to 4 days of salary paid at the legal minimum wage. These findings are important, in light of recent studies on health financing in Guatemala, which have demonstrated that out-of-pocket expense-especially spending on pharmaceuticals in the private exchange market—is the most rapidly growing segment of health care expenditure [22]. Although in theory these medications might be provided through the formal health system at reduced cost, we know from data on other types of medications, such as contraceptives and essential medications for children [29,30], that supply line breaks are endemic in the formal system. A formal study of the marketization and availability of medications for diabetes and other NCDs would be of great interest to confirm and expand our preliminary findings here.

Also with respect to costs, we were surprised to discover that several informants reported higher costs occasioned by traditional treatments and naturopathic healers when compared to allopathic remedies. For example, in other settings, it has been commonly demonstrated that traditional healers are often employed in part because of the associated cost savings [39,40]. One possible explanation may be the very recent boom in the highly profitable complementary and alternative medicine market in Guatemala, which is bleeding over into traditional healing venues [41], but this is an issue that deserves follow-up research to define more concretely.

The quantitative analysis derived from our review of medical charts from first-time medical consultations in our rural primary care clinic provides the most detailed look at clinical characteristics and modifiable risk factors in an indigenous population in Guatemala to date. Among modifiable risk factors, low rates of tobacco and alcohol usage are consistent with known differences in usage rates between both indigenous and non-indigenous Guatemalans as well as males and females [42]. Interestingly, rates of abnormal BMI (BMI ≥ 25) were quite high at 89%, which is even higher than the rate of 77% recently identified in an urban non-indigenous Guatemalan population; at the same time, however, rates of hypertension were nearly two-thirds less [12]. These findings may suggest differential vulnerabilities to risk factors for various non-communicable diseases, as have been widely documented in other indigenous populations [43]. However, further careful research will be needed to explore these preliminary findings.

Finally, our chart review allowed us to explore two markers of disease control and end organ damage (glycosylated hemoglobin and serum creatinine). As shown in Table 1, whether using either more or less stringent criteria for glycosylated hemoglobin [44,45], the majority of individuals had poor control. Comparative data on glycemic control in Latin America are limited, although one multisite study has also concluded that the majority of individuals across the region do not achieve glycemic targets [46]. As such, therefore, our finding here does not provide any quantitative insight into the differential effects of indigeneity on diabetes control; however, it does provide a baseline indicator against which to gauge the success of future diabetes-related programming. Finally, the prevalence of renal insufficiency in our population, defined as a creatinine of greater than 1.5 mg/dl, was only 3.2%. No studies are available for comparison, since the available data on kidney disease in Guatemala and Latin America as a region are largely derived from the population with end-stage renal disease [47]; however, our data provide an initial estimate of kidney disease in our population, which can serve as a starting point for future work.

### Study limitations

This study has several weaknesses that affect the generalizability of the results. With regard to the structured interview sample, those who expressed willingness to participate may differ from the broader population in terms of attitudes towards their health and participation in research. Furthermore, although our statistical comparison of the chart review and interview samples suggests that the quantitative and qualitative results of this study can at least be interpreted together as a whole, both are still derived from a small convenience sample from a single clinic. This convenience sample almost certainly differs in significant ways from the wider indigenous diabetic population. In particular, most patients present to the clinic several years after diagnosis, often for a third or fourth medical opinion; as such, our sample likely exhibits higher rates of comorbidities and risk factors than the general diabetic population. Second, the proportion of females in our sample is higher than the expected prevalence of diabetes in females in Guatemala [9], likely reflecting bias in recruitment methods at the clinic and limiting generalizability of findings to male diabetics. Finally, the sample consists almost exclusively of speakers of the Kaqchikel language; therefore, findings may not be generalizable to speakers of other Mayan languages in Guatemala.

An additional limitation of our study is the way the researchers’ identities may have impacted the interviews and data collected. The interviewers (AC and MG) are both non-Maya women from the United States, who were not known to the study population prior to the interviews. Issues of rapport and trust with investigators may have limited the topics that interviewees were willing to discuss with the researchers; however, at the same time, women interviewees may have felt more comfortable discussing social vulnerabilities related to gender with the interviewers because they too, were women.

Our quantitative analysis is limited by its reliance on health workers’ accurately reporting and entering of data into the electronic record. Among the patient records analyzed, the low proportion of missing variables (Table 1) is reassuring, but this does not exclude the possibility that additional patients were either not entered entirely or had inadequately coded problem and prescription lists, which could have led to missed records based on the range of keywords we used to extract data. Finally, this study assessed prescription rates of medications only, but did not validate medication consumption rates nor distinguish between generic and brand-name pharmaceuticals.

## Conclusions

Major implications of this study for practice and diabetes management revolve around our findings about indigenous diabetics’ economic barriers to treatment, language preferences, and family-based social supports. First, costs of medication and consultations represented significant barriers to treatment for many diabetics, typically associated with intermittent pharmaceutical use and a lack of continuity of care. Diabetes care models that are affordable to indigenous patients are needed. As one step in this direction, we are currently evaluating the effect of a cost-free medication initiative on outcomes within our own clinical program. The second finding of this study with programmatic implications is the strong preference for speaking Mayan languages, rather than Spanish, within this study sample. Diabetics who are speakers of indigenous languages in Guatemala will benefit from delivery of diabetes education and care in their native languages. Currently, in collaboration with the Institute for Nutrition of Central America and Panamá, we are working on a Kaqchikel language diabetes education initiative, which will be evaluated for its effects on patient satisfaction, regimen adherence, and disease outcomes. Lastly, the finding that family members significantly influence diabetics’ experiences of treatment and dietary practices indicates a need for social and educational interventions geared towards families, rather than just individual patients.

Since this study involves a small preliminary sample, directions for future research in Guatemala should also include more in-depth ethnographic investigation about how cultural beliefs and practices, as well as social relations, impact care among indigenous diabetics. For example, further research might explore the dynamics of caregiving within indigenous families with diabetic members, attending particularly to how the social positions of diabetic patients within their households (e.g., as home owner, in-law, parent vs. child, etc.) affect disease management and outcomes.

## Abbreviations

BMI: body mass index; MOH: Ministry of Health; NCD: Noncommunicable disease; NGO: Nongovernmental organization; USD: United States dollars.

## Competing interests

All authors are either volunteer or paid staff members of the study site sponsor, Wuqu’ Kawoq.

## Authors’ contributions

AC collected and analyzed qualitative data and helped draft the manuscript. MG collected and analyzed qualitative data. CB collected and analyzed medical records data. PR conceived study, participated in its design and coordination, served as contest reviewer for qualitative data, and drafted the manuscript. All authors read and approved the final manuscript.

## Pre-publication history

The pre-publication history for this paper can be accessed here:

http://www.biomedcentral.com/1472-6963/12/476/prepub
